# Transfer of newborns to neonatal care unit: a registry based study in Northern Tanzania

**DOI:** 10.1186/1471-2393-11-68

**Published:** 2011-10-04

**Authors:** Blandina T Mmbaga, Rolv T Lie, Gibson S Kibiki, Raimos Olomi, Gunnar Kvåle, Anne K Daltveit

**Affiliations:** 1 Kilimanjaro Christian Medical Centre and Kilimanjaro Christian Medical College, P.O Box 3010, Moshi, Tanzania; 2 Department of Public Health and Primary Health Care, University of Bergen, P.O Box 7804, 5020 Bergen, Norway; 3 Centre for International Health, University of Bergen, P.O Box 7804, 5020 Bergen, Norway; 4 Norwegian Institute of Public Health, Division of Epidemiology, Oslo, Norway; 5 Kilimanjaro Clinical Research Institute, P.O Box 2236 Moshi, Tanzania

## Abstract

**Background:**

Reduction in neonatal mortality has been slower than anticipated in many low income countries including Tanzania. Adequate neonatal care may contribute to reduced mortality. We studied factors associated with transfer of babies to a neonatal care unit (NCU) in data from a birth registry at Kilimanjaro Christian Medical Centre (KCMC) in Tanzania.

**Methods:**

A total of 21 206 singleton live births registered from 2000 to 2008 were included. Multivariable analysis was carried out to study neonatal transfer to NCU by socio-demographic factors, pregnancy complications and measures of the condition of the newborn.

**Results:**

A total of 3190 (15%) newborn singletons were transferred to the NCU. As expected, neonatal transfer was strongly associated with specific conditions of the baby including birth weight above 4000 g (relative risk (RR) = 7.2; 95% confidence interval (CI) 6.5-8.0) or below 1500 g (RR = 3.0; 95% CI: 2.3-4.0), five minutes Apgar score less than 7 (RR = 4.0; 95% CI: 3.4-4.6), and preterm birth before 34 weeks of gestation (RR = 1.8; 95% CI: 1.5-2.1). However, pregnancy- and delivery-related conditions like premature rupture of membrane (RR = 2.3; 95% CI: 1.9-2.7), preeclampsia (RR = 1.3; 95% CI: 1.1-1.5), other vaginal delivery (RR = 2.2; 95% CI: 1.7-2.9) and caesarean section (RR = 1.9; 95% CI: 1.8-2.1) were also significantly associated with transfer. Birth to a first born child was associated with increased likelihood of transfer (relative risk (RR) 1.4; 95% CI: 1.2-1.5), while the likelihood was reduced (RR = 0.5; 95% CI: 0.3-0.9) when the father had no education.

**Conclusions:**

In addition to strong associations between neonatal transfer and classical neonatal risk factors for morbidity and mortality, some pregnancy-related and demographic factors were predictors of neonatal transfer. Overall, transfer was more likely for babies with signs of poor health status or a complicated pregnancy. Except for a possibly reduced use of transfer for babies of non-educated fathers and a high transfer rate for first born babies, there were no signs that transfer was based on non-medical indications.

## Background

Progress on United Nations' Millennium Development Goal 4 (MDG4) to reduce the under-five mortality has been slower than anticipated due to high neonatal mortality in developing countries. Worldwide, about 4 million neonatal deaths occur each year, of these three quarter occur in the first week of life with the highest risk at the first day of life [1]. Estimated neonatal mortality in Tanzania is about 35 per 1000 live births, and neonatal deaths are estimated to account for 28% of the under-five mortality [2]. Both the infant mortality rate and the under -five mortality rate have decreased from 1990 to 2004; by 31% (from 99 to 68 deaths per 1000 live births) and 24% (from 147 to 112 deaths per 1000 live births), respectively. This decline was, however, observed for post-neonatal mortality only, while neonatal as well as maternal mortality remained unchanged [2,3]. Adequate neonatal care may therefore be an important factor for continued improvement. Socio-economic deprivations are known to cause poor perinatal outcome such as neonatal care admission [4-7], low birth weight [8-10] and increased perinatal mortality [10-13]. A review of international evidence in socio-economic inequalities in childhood mortality in low and middle income countries showed higher childhood mortality in low socio-economic groups within each country [14]. Absolute inequalities were found to be higher for infant mortality than for child mortality. It was also estimated that 20-25% of under-five mortality inequalities arise in the neonatal period [14]. Making sure that health care is provided independent of social status is important for overall improvement in health.

Most studies on neonatal health in developing countries have focused on mortality rather than morbidity. However, in order to reduce neonatal mortality it is also of importance to consider factors associated with neonatal morbidity. Transfer of babies to neonatal care unit (NCU) may represent an indicator of morbidity that can be used for designing and implementing interventions aimed at improving health and increasing neonatal survival. Although previous studies have reported on the relationship of socio-demographic, maternal, or neonatal factors with neonatal admission [5,6,15], the combined effect of socio-demographic, maternal health factors and neonatal factors in relation to admission to NCU has not been well explored.

Referral in pregnancy and child birth can be categorised as self-referral or referral performed by health workers [16]. Self-referral implies that a woman (perhaps with the help of her family) seeks care at a health centre or a hospital. A study of 415 maternity admissions in Tanzania found that about 70% of the admissions could be categorized as self-referrals [16].

The presence of a NCU at the hospital gives an opportunity for all at risk babies to be admitted and managed by a paediatrician. The paediatric department at KCMC has established guidelines for care and management of newborns based on the condition of the newborn. Decision for transfer is usually done by midwives or a paediatrician based on the condition of the newborn; low Apgar score, prematurity, birth weight <1800 or birth weight >4000 g, congenital malformation and suspected infection. In addition, some obstetric conditions may necessitate baby transfer because they could represent a risk to the newborn. When a pregnancy complication indicates that the baby needs to be seen by a paediatrician, the paediatrician is informed in advance and attends the delivery to take care of the newborn in the labour ward or in NCU if transfer is necessary. The parents are usually informed about the reason for babies transfer but they are not asked for decision. Although KCMC is a private hospital, payment for the hospital bill is not considered as initial criteria for transfer or management of admitted newborns, therefore, all admitted babies receive same quality of care irrespective of the social background. The social welfare department within the hospital usually takes care of the hospital bills for families unable to pay.

The aim of our analysis was to estimate the influence of social background, pregnancy-related conditions and the condition of the newborn in relation to neonatal transfer to NCU. We explore these associations in a structured series of analyses, expecting most of the associations to be explained by the condition of the newborn. First, we expect social conditions to impact the likelihood of transfer by their effects on pregnancy complications and the condition of the newborn. Then we expect pregnancy complications to impact the likelihood of transfer by their effects on the condition of the newborn. Deviations from these expectations will appear as residual effects of social background and pregnancy complications after we adjust for the condition of the newborn. Such deviations will be inspected further since they could represent priority-settings or clinical judgment that incorporates social background or the background history of the delivery.

## Methods

### Setting

This study was done at Kilimanjaro Christian Medical Centre (KCMC) in Northern Tanzania. The hospital is a zonal hospital serving more than 13 million people from 4 regions namely; Kilimanjaro, Arusha, Tanga and Manyara. We established a cohort of babies based on records from the Medical Birth Registry comprising all deliveries at the hospital from July 2000 to September 2008 and followed the cohort in a registry of neonates transferred from the labour ward to NCU. The KCMC Medical Birth Registry system was established in 1999 as a collaboration between Kilimanjaro Christian Medical College, Tumaini University and the University of Bergen, Norway. The annual number of deliveries is around 3000 of which nearly two thirds are from urban area. Approximately 10-15% of the neonates are transferred to NCU for observation and management.

**Figure 1:**
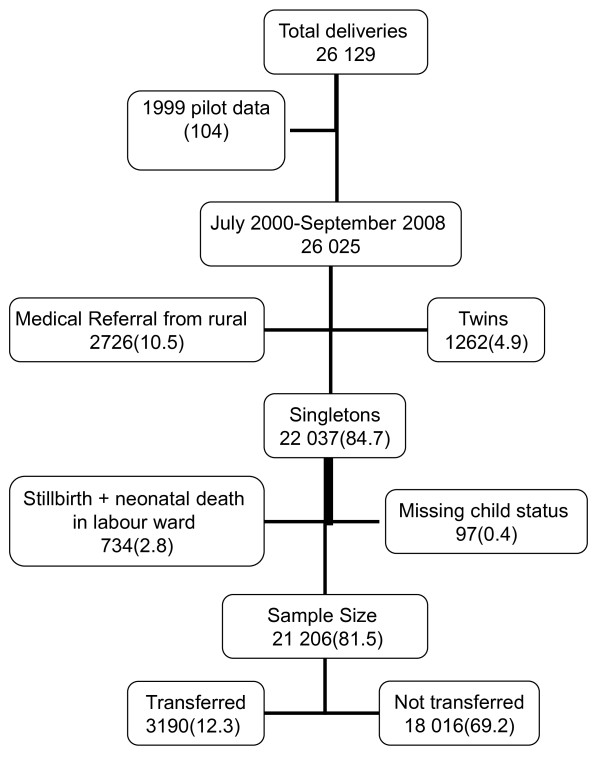
**Schematic diagram of the study population and sample sizes**. Number in bracket represents proportion of neonates included or excluded from the study based on the July 2000-September 2008 study cohort (21 206).

A total of 26 025 births were recorded in the Medical Birth Registry from July 1^st ^2000 to September 30^th ^2008. We excluded multiple deliveries, stillbirths, neonatal deaths in labour ward and neonates with missing child status record after delivery (Figure 1). In order to obtain a study group that reflected the general population, we excluded deliveries where mothers residing in rural areas had been referred for delivery at KCMC for medical reasons. Women residing in Moshi urban were not excluded since they could have delivered at KCMC anyway. KCMC is located in Moshi Urban and that 50% of the deliveries at KCMC are from Moshi Urban district [11]. We finally analyzed a total of 21 206 singleton live births.

### Data collection

Information from all mothers who delivered at KCMC were collected within the first 24 hours after delivery. Trained midwife nurses conducted the interviews on a daily basis with all eligible subjects using a standardized questionnaire. A verbal consent was obtained from the participants prior to the interview. Mothers also provided their antenatal visit card for more information such as date of first ANC visit, immunization history, malaria prophylaxis, drugs, illnesses recorded during follow up, weight at first ANC visit, number of ANC visits, as well as referral to ANC (self-referred or referred by health worker).

Information in the birth registry includes maternal health conditions before and during pregnancy, parents' socio-demographic characteristics, complications during labour and delivery, and information on the newborn; sex, gestational age, birth weight, Apgar score, and child status in four categories: 1) live born 2) live born transferred to NCU 3) neonatal death in labour ward, 4) stillborn.

The paediatric registry form was recorded in the NCU for all neonates who were transferred. The neonatal registry includes information on primary reasons for transfer, management, and discharge/death diagnoses. The two databases were linked using the unique child identification number, the mother's hospital registration, and the newborn's birth registration number.

### Variable definition

Transfer to NCU was the main outcome. Independent variables include socio-demographic characteristics including maternal, paternal and environmental factors, maternal health conditions before and during pregnancy, and complications during labour and delivery, as well as condition of the newborn (Tables 1, 2 and 3).

**Table 1:** Transfer to neonatal care unit (n = 3190) among 21 206 live-born according to socio-demographic factors

Risk factors	Number live-born deliveries	Proportion (%) live-born babies transferred to NCU	RR (95% CI)	p-value
**Maternal factors**^x^				
Maternal age (years)				0.106
Under 18	480	17.9	1.2 (1.0-1.5)	
18-25	8328	14.5	1.0	
26-35	10 272	15.3	1.0 (1.0-1.1)	
Over 35	2070	15.7	1.0 (1.0-1.2)	
Mother's tribe				0.032
Chagga	12 311	14.5	1.0	
Pare	2496	16.2	1.0 (1.0-1.2)	
Others	6355	15.6	1.0 (1.0-1.2)	
Marital status				<0.0001
Married	19 016	14.6	1.0	
Single	2086	18.6	1.3 (1.2-1.4)	
Birth order				<0.0001
1^st ^Child	8220	16.4	1.2 (1.1-1.3)	
2^nd ^Child	5985	13.4	1.0	
3^rd ^Child	3287	16.5	1.0 (0.9-1.1)	
4^th ^or more	3714	13.5	1.2 (1.1-1.4)	
Mother's education				0.060
No education	348	17.8	1.2(1.0-1.7)	
Primary	12 990	15.3	1.0(1.0-1.1)	
Sec/higher	7819	14.4	1.0	
Mother's occupation				<0.0001
Professional	3355	14.3	1.0	
Business	4821	14.8	1.0 (0.9-1.2)	
Service	1538	15.4	1.1 (0.9-1.2)	
Farmer	4003	16.3	1.1 (1.0-1.3)	
Housewife	5401	15.4	1.1 (1.0-1.2)	
Others	1955	13.3	1.0 (0.8-1.1)	
Body height (cm)				<0.0001
<150	1505	18.3	1.4 (1.2-1.5)	
150+	18 342	14.1	1.0	
BMI (kg/m^2^)				0.013
<18.5	1277	14.1	1.1 (0.9-1.2)	
18.5-24.9	4787	14.8	1.0	
25-29.9	6793	13.3	1.1 (1.0-1.2)	
30+	1496	16.2	1.2 (1.1-1.4)	
Genital mutilation				0.086
Yes	4752	15.8	1.0 (0.9-1.0)	
No	16 389	14.8	1.0	
Drinking in pregnancy				0.033
Yes	8278	14.4	1.0	
No	12 882	15.4	1.1 (1.0-1.2)	
**Paternal factors**^x^				
Father's age (years)				0.003
Under 26	3002	16.7	1.1 (1.1-1.3)	
26-35	11 794	14.6	1.0	
36-45	5428	14.5	1.0 (0.9-1.1)	
Over 45	827	17.7	1.2 (1.0-1.4)	
Father's tribe				0.004
Chagga	11 157	14.2	1.0	
Pare	2463	16.0	1.1(1.0-1.3)	
Others	7451	15.8	1.1(1.0-1.2)	
Father's Occupation				<0.0001
Professional	4483	14.0	1.0	
Business	6798	14.8	1.1 (1.0-1.2)	
Service	4321	15.0	1.1 (1.0-1.2)	
Farmer	2070	18.7	1.3 (1.2-1.5)	
Skilled	2808	14.1	1.0 (0.9-1.1)	
Others	643	15.9	1.1 (0.9-1.4)	
Father's education				0.013
No education	110	20.9	1.5 (1.0-2.1)	
Primary	10 563	15.5	1.1 (1.0-1.2)	
Sec/higher	10 446	14.4	1.0	
**Environmental factors**^x^				
Type of toilet				0.006
Pit latrine	12 515	15.6	1.1 (1.0-1.2)	
Flush	8608	14.2	1.0	
Source of water				0.030
Tap water	19 555	14.9	1.0	
Well	459	16.6	1.1 (0.9-1.4)	
River	432	19.7	1.3 (1.1-1.6)	
Spring	644	16.0	1.1 (0.9-1.3)	
Boil drinking water				<0.0001
Yes	6672	13.3		
No	14 449	15.8	1.2 (1.1-1.3)	

x-The total in some variables does not sum to 21 206 due to missing data

**Table 2:** Transfer to neonatal care unit (n = 3190) among 21 206 live-born according to maternal health conditions

Risk factors	Number live-born deliveries	Proportion (%) live-born babies transferred to NCU	RR (95% CI)	p-value
**Before pregnancy^a^**				
Medication regular	493	18.9	1.3 (1.1-1.5)	0.013
Diabetes	49	69.4	4.7 (3.9-5.6)	<0.0001
Hypertension	143	21.7	1.5 (1.1-2.0)	0.021
Epilepsy	64	25.0	1.7 (1.1-2.6)	0.026
Gyn. Disease	1122	17.5	1.2 (1.0-1.4)	0.020
Lung disease	1950	16.6	1.1 (1.0-1.2)	0.040
Malaria	12 258	14.9	1.0 (0.9-1.1)	0.899
Anaemia	406	17.0	1.2 (0.9-1.5)	0.267
Tuberculosis	77	18.2	1.3 (0.7-2.2)	0.703
				
**During pregnancy^a^**				
No ANC attendance	137	34.3	2.3 (1.8-2.9)	<0.0001
Referred to ANC^§^	2284	21.0	1.5 (1.4-1.6)	<0.0001
ANC < 5 visits	13 168	16.2	1.3 (1.2-1.4)	<0.0001
Anaemia	449	18.3	1.2 (1.0-1.5)	0.050
Gestational Diabetic	17	47.1	3.1 (1.9-5.2)	<0.0001
Hypertension	72	30.6	2.0 (1.4-2.9)	<0.0001
Preeclampsia	711	32.1	2.2 (2.0-2.5)	<0.0001
Eclampsia	27	70.4	4.7 (3.7-6.0)	<0.0001
Bleeding	239	23	1.5 (1.2-2.0)	<0.0001
Malaria	4314	14.4	1.0 (0.9-1.0)	0.167
Tuberculosis	414	15.0	1.0 (0.8-1.3)	0.969
HIV infection	784	16.1	1.0 (0.8-1.1)	0.528
				
**Complications^a^**				
Abruptio placenta	29	65.5	4.4 (3.4-5.7)	<0.0001
PROM	468	54.7	3.9 (3.5-4.2)	<0.0001
Bleeding >500 mls	36	33.3	2.2 (1.4-3.5)	0.001
Placenta previa	51	45.1	3.0 (2.2-4.1)	<0.0001
Caesarean section	6472	24.2	2.3 (2.2-2.5)	<0.0001
Other Vaginal delivery	317	33.4	3.2 (2.7-3.8)	<0.0001
Other unspecified	373	24.7	1.7 (1.4-2.0)	<0.0001

a- Numbers for reference categories not given, each variable had complete data

§- First ANC visit triggered by health workers

**Table 3:** Transfer to neonatal care unit (n = 3190) among 21 206 live-born according to newborn health conditions

Risk factors^x^	Number live-born deliveries	Proportion (%) live-born babies transferred to NCU	RR (95% CI)	p-value
Birth weight (g)				<0.0001
500-1499	173	95.4	9.8 (9.3-10.4)	
1500-2499	1652	41.5	4.3 (4.0-4.6)	
2500-3999	18 607	9.7		
4000-6000	714	69.7	7.2 (6.7-7.7)	
Apgar score 5 min				<0.0001
<7	442	91.9	6.9 (6.6-7.2)	
7+	20 590	13.4		
Gestation age (weeks)				<0.0001
25-33	447	70.5	5.6 (5.2-6.1)	
34-36	1401	27.3	2.2 (2.0-2.4)	
37+	17 603	12.5	1.0	
Presentation				<0.0001
Cephalic	20 862	14.8	1.0	
Breech	238	28.2	1.9 (1.6-2.3)	
Transverse	28	21.4	1.5 (0.7-3.0)	
Sex				<0.0001
Male	10 904	16.6	1.2 (1.2-1.3)	
Female	10 162	13.3	1.0	

x-The total in some variables does not sum to 21 206 due to missing data

### Data analysis

Data were analyzed using Statistical Package for Social Science (SPSS) program Version 15.0 for Windows (SPSS 15.0 Chicago Inc. III, USA). Cross tabulations and generalized linear models were used to obtain relative risks (RR) and corresponding 95% confidence intervals. From the bivariate analyses we present all variables with p-value less than 0.1, which were then entered into the multivariable analysis. Three steps were involved in the multivariable analysis. In the first step (model A) all socio-demographic factors and maternal health condition before pregnancy were included. In the second step (model B) we included all variables in step one as well as pregnancy and labour-related conditions. In the third and final step (model C), we included all variables in step two as well as neonatal conditions. We used Poisson regression with robust variances to obtain a valid confidence interval when a log-binomial analysis failed to converge [17]. A priori we also considered some maternal conditions to be important and included in the final analysis, these were hypertensive conditions (preeclampsia, eclampsia and abruption placenta) and diabetes (pre-gestational or gestational).

### Ethical approval

The birth registry at Kilimanjaro Christian Medical Centre obtained ethical clearance from the Tanzania Ministry of Health, Institute of Science and Technology, from the Norwegian National ethics committee and from the Kilimanjaro Christian Medical College (KCM-College) research ethics committee in 1999. The protocol for this study was approved by KCM-College research ethics committee, with certificate no. 333 of 15^th ^July 2010.

## Results

A total of 21 206 live-born singletons were analysed. The majority of the mothers were married (89.7%), were residing in urban areas (61.9%), had primary school education (61.3%), and belonged to the *Chagga *tribe (58.2%). Mean maternal age at child birth was 27.4 (SD = 6.1) years, and 38.8% of the mothers had their first born child. Mean maternal pre-pregnancy weight and height were 62.7 (SD = 12.5) kilograms and 160.0 (SD = 6.7) centimetres, respectively. The mean number of antenatal care visits per women was 5 (SD = 2.1). Mean gestational age and birth weight were 39.1 (SD = 2.5) weeks and 3090 (SD = 544 grams), respectively.

A total of 3190 (15%) were transferred to NCU. Descriptive associations between transfer and socio-demographic, pregnancy-related and neonatal factors are shown in Tables 1, 2 and 3.

### Socio-demographic characteristics and pre pregnancy conditions

After mutual adjustment of the socio-demographic and maternal pre-pregnancy health factors, most of the factors remained associated with neonatal transfer (Table 4; model A). First born babies and fourth or later born babies (RR 1.3; 95% CI: 1.2-1.4 and 1.2; 95% CI: 1.0-1.3, respectively) were shown to have a high risk of being transferred compared with second born babies. Babies of single mothers were more likely to be transferred compared to babies of married mothers (RR 1.3; 95% CI: 1.1-1.5). Both maternal overweight and obesity increased the risk of babies transfer. Babies born from families who do not boil water for drinking had increased risk of being transferred to NCU (RR 1.2; 95% CI: 1.1-1.3).

**Table 4:** Linear regression model risk factors for neonatal transfer to neonatal care unit

	Model A^a^	Model B^a^	Model C^a^
	
Risk factors	RR (95%CI)	RR (95%CI)	RR (95%CI)
**Pre-pregnancy factors**			
Maternal age (Ref. 18-25 years)			
Under 18 years	1.0 (0.8-1.4)	1.0 (0.8-1.4)	0.9 (0.7-1.2)
26-35 years	1.3 (1.1-1.4)**	1.2 (1.1-1.3)**	1.2 (1.1-1.3)**
Over 35 years	1.1 (0.9-1.3)	1.0 (0.8-1.3)	1.0 (0.8-1.2)
Birth order (Ref. 2^nd ^child)			
1^st ^child	1.3 (1.2-1.4)**	1.3 (1.2-1.5)**	1.4 (1.2-1.5)**
3^rd ^child	1.0 (0.8-1.1)	1.0 (0.9-1.1)	1.0 (0.9-1.1)
4^th ^or more	1.2 (1.0-1.3)	1.3 (1.1-1.4)**	1.1 (1.0-1.3)
Body mass index(Ref 18.5-24.9)			
Underweight (<18.5)	1.0 (0.9-1.2)	1.0 (0.9-1.2)	1.0 (0.9-1.2)
Overweight (25-29.9)	1.2 (1.1-1.3)**	1.2 (1.0-1.3)**	1.1 (1.0-1.2)
Obesity(30+)	1.3 (1.1-1.5)**	1.2 (1.1-1.4)**	1.1 (1.0-1.3)
Single marital status	1.3 (1.1-1.5)**	1.2 (1.1-1.4)*	1.2 (1.0-1.3)*
Body height <150 cm	1.2 (1.1-1.4)*	1.0 (0.9-1.2)	1.1 c(0.9-1.2)
Paternal age (Ref 26-35 years)			
Under 26 years	1.2 (1.0-1.3)	1.1 (1.0-1.3)	1.2 (1.0-1.3)*
36-45 years	1.0 (0.9-1.1)	0.9 (0.8-1.0)	0.9 (0.8-1.1)
Over 45 years	1.1 (0.9-1.4)	1.1 (0.9-1.4)	1.1 (0.9-1.3)
Father's education (Ref sec/high)			
No education	1.2 (0.7-2.3)	0.8 (0.5-1.5)	0.5 (0.3-0.9)*
Primary school	1.0 (0.9-1.1)	1.0 (0.9-1.1)	1.0 (0.9-1.1)
Pre-gestational diabetic	4.4 (3.3-5.8)**	3.5 (2.6-4.7)**	1.6 (0.7-3.3)
Maternal Lung disease	1.2 (1.1-1.4)**	1.2 (1.1-1.4)**	1.2 (1.0-1.3)*
Maternal Epilepsy	1.6 (1.0-2.6)	1.9 (1.2-2.9)**	1.4 (0.9-2.2)
Not boiling drinking water	1.2 (1.1-1.3)**	1.1 (1.0-1.3)**	1.1 (1.0-1.2)

**Pregnancy, labour and delivery**			
Mother referred to ANC^§^	**-**	1.3 (1.1-1.4)**	1.2 (1.0-1.3)*
ANC < 5 visits	-	1.3 (1.2-1.4)	1.2 (1.1-1.3)**
Gestational Diabetic	-	1.4 (0.6-3.4)	1.4 (0.5-4.5)
Hypertension		1.5 (0.9-2.4)	1.2 (0.7-1.9)
Preeclampsia		2.0 (1.7-2.3)**	1.3 (1.1-1.5)**
Eclampsia	-	2.8 (1.7-4.4)**	0.9 (0.6-1.6)
Abruptio placenta	-	2.6 (1.6-4.1)**	1.1 (0.7-1.8)
Premature rupture of membrane	-	2.9 (2.6-3.4)**	2.3 (1.9-2.7)*
Caesarian section	-	2.1 (1.9-2.3)**	1.9 (1.8-2.1)**
Other vaginal delivery	-	2.9 (2.3-3.6)**	2.2 (1.7-2.9)**
Other unspecified complications	-	1.8 (1.4-2.3)**	1.5 (1.2-1.9)**

**Neonatal factors**			
Birth weight >4000 g	-	-	7.2 (6.5-8.0)**
Birth Weight 1500-2500 g	-	-	2.8 (2.5-3.1)**
Birth weight <1500 g	-	-	3.0 (2.3-4.0)**'
Gestational age below 34 weeks	-	-	1.8 (1.5-2.1)**
Gestational age 34-36 weeks	-	-	1.3 1.3 (1.1-1.5)**
Five minutes Apgar score <7	-	-	4.0 (3.4-4.6)**
Male sex			1.2 (1.1-1.3)**

*p-value less than 0.05

**p-value less than 0.01

^a ^In each step variables entered were all which had p-value of < 0.1 in univariable analysis including maternal age although p-value was slightly above 0.1 (0.106). The lowest risk category in each group was used as a reference. Results are presented for all variables which were significant at least once in any of the three steps.

Model A, first step; adjusted for pre pregnancy factors

Model B, second step; variables in model A plus conditions in pregnancy, labor and delivery

Model C, third step; variables in model B plus neonatal factors

§- First ANC visit triggered by health workers

Pre-gestational diabetes mellitus was strongly associated with neonatal transfer to NCU (RR 4.4; 95% CI: 3.3-5.8). A history of acute or chronic lung disease other than tuberculosis showed a weaker association (RR 1.2; 95% CI: 1.1-1.4).

### Pregnancy, labour and delivery

Factors related to pregnancy, labour and delivery were included in the multivariable model in B. Hypertensive conditions such as eclampsia and preeclampsia (RR 2.8; 95% CI:1.7-4.4 and 2.0; 95% CI: 1.7-2.3, respectively), labour-related complications such as premature rupture of membrane and abruption placenta (RR 2.9; 95% CI: 2.6-3.4 and 2.6; 95% CI: 1.6-4.1, respectively), and other vaginal delivery (i.e. breech, vacuum or forceps) and caesarean section delivery (RR 2.9; 95% CI: 2.3-3.6 and 2.1; 95% CI: 1.9-2.3, respectively) were all associated with transfer (Table 4; model B). Gestational diabetes increased the risk of babies transfer by 40% although not statistically significant. Referral to ANC and few ANC visits were also found to be important predictors of neonatal transfer to NCU (RR 1.3; 95% CI: 1.1-1.4 and 1.3; 95% CI: 1.2-1.4), respectively.

Significant factors in model A continued to be independent predictors for neonatal transfer also in model B, except for maternal body height below 150 cm. However, addition of variables in model B slightly reduced the relative risk for most factors.

### Neonatal factors

In model C, neonatal factors were added into the multivariable model. All the selected neonatal factors were significantly associated with transfer to NCU, with the highest relative risks being for birth weight above 4000 g (RR 7.2; 95% CI: 6.5-8.0) and five minutes Apgar score below 7 (RR 4.0; 95% CI: 3.4-4.6) (Table 4; model C).

After inclusion of the neonatal factors, some pre-pregnancy factors, such as women giving birth to their first babies (RR 1.4; 95% CI: 1.2-1.5), maternal age 26-35 years (RR 1.2; 95% CI: 1.1-1.3), and single marital status (RR 1.2; 95% CI: 1.0-1.3) were still significantly associated with neonatal transfer. Lack of paternal education (RR: 0.5; 95% CI: 0.3-0.9) was negatively associated with transfer to NCU. Birth to fourth or later born babies, maternal overweight or obesity, pre-gestational diabetes and epilepsy were no longer significantly associated with neonatal transfer.

## Discussion

In this registry based study from a tertiary hospital in Tanzania, we identified patterns of neonatal transfer to NCU. In a three-step analysis we studied socio-demographic factors, maternal health factors, and neonatal factors in relation to transfer. A particular aim was to assess whether socio-demographic factors were related to transfer to NCU beyond their association with well-defined medical risks. The analyses showed that neonatal factors by far had the strongest association with neonatal transfer, but that pre-pregnancy and pregnancy factors were also independently associated with transfer.

The incidence of neonatal transfer in this study was 15%, which is slightly higher than reported in previous studies both from developed [4,5] and developing countries [7,18].

### Neonatal factors

The studied neonatal factors included classical risk factors for morbidity and mortality, such as birth weight, preterm delivery, Apgar score and sex, and were as expected strongly related to neonatal transfer. Although the causal effect of birth weight is controversial [19] low birth weight is a good predictor of need for neonatal care. Low birth weight has been proposed to contribute to 40-80% of neonatal morbidity and mortality [20,21]. Preterm delivery is estimated to account for 28% of all neonatal deaths [20].

We also found a very high admission rate of newborns with a birth weight above 4000 g. Fetal macrosomia is associated with obstetric complications and neonatal morbidity such as injuries, respiratory distress and hypoglycaemia. Observation for transient or persistent hypoglycaemia is a common reason for admission of high birth weight babies to NCU [22]. At KCMC, such babies will be discharged within 24 hours if there is no risk of persistent hypoglycemia and the blood glucose level is normal. The outcome is in general good for these babies, and one may speculate whether observation without transfer to NCU for many of these babies would represent a better use of resources.

In general, male neonatal morbidity exceeds female morbidity, partly due to a higher occurrence of preterm birth and other neonatal risk factors [23]. The male-to-female ratio of transfer 1.24, declining to 1.18 in the adjusted analyses, corresponds well with the established higher risk in males, and does not indicate a difference in care according to infant sex.

### Pregnancy, labour and delivery

Risk of neonatal transfer was high in mothers with preeclampsia, eclampsia and abruption placenta, however no or weak effects were observed after inclusion of neonatal factors in the model. Hypertensive conditions in pregnancy are associated with preterm birth and low birth weight [15,24-27], and many cases of abruption placenta occur at a low gestational age, which explain the indirect association between these complications and neonatal transfer. The direct cause of transfer would be the preterm birth.

Other conditions, such as premature rupture of membrane, caesarean section and operative vaginal delivery, showed a high risk of neonatal transfer also after accounting for the neonatal condition of the baby. The high rate of transfer for babies born with mothers having PROM is similar to what is reported elsewhere [15]. Premature rupture of the membrane (PROM) is associated with preterm delivery and low birth weight [15,27]. A previous study at KCMC reported a high prevalence (38%) of low birth weight babies after PROM [27]. Such babies are at higher risk of developing neonatal infection. Antibiotic prophylaxis given to mothers with PROM has shown to reduce risk of infection in the newborn [28,29]. The high transfer rate after PROM in our data is likely to be explained by the fact that a majority of mothers with a history of PROM did not receive antibiotic prophylaxis prior to delivery, due to late arrival to the centre.

Mothers with less than five antenatal care visits were more likely to have their baby transferred and this association persisted after we took into account our measures of the condition of the newborn. Amount of antenatal care plays a role in neonatal outcome [30-32], and each additional ANC visit has previously been found to offer a protective effect on neonatal outcome [31]. When the mother had been referred for antenatal care, however, the risk of transfer was increased.

### Pre pregnancy factors

Among diseases that the mothers had before the pregnancy, only lung disease remained significantly associated with neonatal transfer when pregnancy conditions and neonatal conditions were accounted for (Table 4, model C). Pre-gestational diabetes was strongly related to transfer in models A and B, but the association disappeared after accounting for the neonatal conditions in model C. Noteworthy, gestational diabetes had a weak and non-significant association with transfer, and the relative risk was not affected by adjustment for neonatal factors. The low risk of transfer in babies born to mothers with gestational diabetes compared to babies of mothers with pre-gestational diabetes is also reported elsewhere [5,15,33].

Women giving birth to their first child and single mothers were more likely to have their baby transferred to NCU, also after accounting for pregnancy conditions and neonatal conditions. Birth to a first child and single motherhood are classical risk factors for neonatal morbidity and mortality [9,10,12,18,34,35]. However, the 40% higher risk of admission for a first born child in the fully adjusted model (model C), is higher than what one would expect according to previous knowledge on morbidity and mortality associated with first delivery. In a previous study from the same hospital, perinatal mortality was not associated with birth order except for a higher perinatal mortality in offsprings of mothers with three or more previous pregnancies [11]. To further elaborate this finding, we performed a regression analysis with a finer categorization of Apgar score. In this model, the parity effect was still statistically significant, however reduced. In a setting with limited obstetric services, the generally higher neonatal stress on first born babies might be even more evident.

In line with previous findings [36-38] we found that overweight and in particular obese mothers had a high risk of having their baby transferred to NCU. Maternal obesity is associated with some pregnancy complications [33,36-40] and overweight or obese mothers are more likely to have high birth weight babies [37,38,40]. A meta-analysis review showed a lower risk of low birth weight among babies of overweight or obese mothers compared to normal weight mothers, however the risk of very low birth weight and extremely low birth weight was increased due to more induced preterm deliveries in overweight or obese mothers [41]. In our data, the association of neonatal transfer associated with maternal overweight and obesity was weakened but still statistically significant after adjustment for pregnancy conditions, however disappeared after adjustment for neonatal conditions. Hence, pregnancy conditions and neonatal conditions seem to be mediators in the association between maternal overweight and neonatal transfer. A similar pattern was seen for mothers of short stature, where an increased risk seen in model A seemed to be linked to a higher rate of pregnancy complications for these mothers.

Drinking unboiled water was one of the factors associated with neonatal transfer. Waterborne disease including diarrhoea and dysentery is prevalent in Tanzania, therefore, it is recommended to boil water for drinking including tap water. In our study 92% of the participants used tap water, however only 31% boiled water for drinking. In a study from Tanzania, lack of boiling water prior to consumption was more common in households with low income, and lack of proper knowledge on the importance of how to handle and store water safely was associated with E.coli occurrence [42]. Both ignorance and poverty might be the major barriers to boiling drinking water.

Lack of paternal education was associated with a low chance of neonatal transfer (RR = 0.5; 95% CI: 0.3-0.9) in the fully adjusted model. Although our results should be interpreted with care due to the low numbers (110 fathers with no education) and a confidence interval close to one, the findings could reflect low focus on neonatal health care in deprived families. A previous study using the same birth registry reported that paternal socio-demographic factors seemed to be more important predictors of perinatal mortality than maternal socio-demographic factors in this area [11]. However, such an interpretation is not compatible with the principle that transfer mechanisms should be unaffected by parental and family influence.

## Strengths and limitations

The study was based on a hospital based birth registry, where data are carefully collected according to standardized procedures, ensuring complete coverage of births on a daily basis including weekends and holidays. Information was collected by designated midwives using a structured questionnaire-based interview, and medical records were used to verify the information from the questionnaire. The sample size was relatively large and enabled us to study many risk factors in relation to neonatal transfer. Hence, the data allowed us to study the relationship between socio-demographic characteristics, maternal health and complications during delivery, and neonatal characteristics, with transfer to neonatal intensive care unit. Selection bias was reduced by excluding all medically indicated referral births from rural areas where the mother would not probably deliver at KCMC if not referred. The excluded cases accounted for 52% of all referrals and 75% of all medical referrals.

About 29% of the deliveries in the Kilimanjaro region occur at home [20], and the study results may not be representative of the entire population within the area. Although women who give birth at the hospital largely differ with respect to socio-demographic status, the socio-demographic variation in the community may be even larger and towards a less privileged population. It is therefore possible that the observed risks are underestimated as compared to the region.

We applied an analytical approach where the various classes of variables were included in regression models through three steps. The purpose of this was to identify which factors that mediated any association with transfer. Our analyses are based on a limited set of variables, and there may be important risk factors of neonatal transfer that we have not been able to account for. Hence, the effects obtained in the models may represent a mixture of effects of the studied factors and effects of factors not accounted for. In particular, our measures of the condition of the newborn were probably too crude to fully account for the clinical judgement of the baby's condition and the need for transfer.

Despite these limitations, we believe that our study, based on structured collection of information with a hospital based design combined with careful considerations of possible biases, represent findings of importance. True population data are difficult to collect in sub-Saharan Africa. Investment in competence building and data collection should start with key hospitals, and efforts should be done to include well-defined populations, in order to generate relevant and representative data to address the important public health issues within the general population.

## Conclusions

Our study has demonstrated the combined effect of socio-demographic, maternal health conditions and neonatal factors in predicting transfer to NCU. The relationship between socio-demographic, maternal health characteristics and neonatal factors observed in this study reflects traditionally known predictors of neonatal morbidity and mortality. As for the pre-pregnancy factors, most of the associations with transfer were accounted for by pregnancy complications and neonatal factors. An exception from this was a possibly reduced use of transfer for babies of non-educated fathers. The potential effect of paternal social status both on neonatal health and on access to health care for mother and baby needs more attention. Another exception that needs to be further explored is the 40% higher rate of transfer among first born babies. With respect to neonatal factors, one might speculate whether the high number of babies above 4000 g transferred to the NCU represents an optimal use of resources, as the outcome of these babies is in general good.

## Competing interests

The authors declare that they have no competing interests.

## Authors' contributions

BTM: Study design, methodology, data analysis and manuscript writing. RTL, GSK, RO, GK, AKD: Study design, methodology, manuscript writing. All authors approved the final manuscript.

## Pre-publication history

The pre-publication history for this paper can be accessed here:

http://www.biomedcentral.com/1471-2393/11/68/prepub
